# Insight Into the Virulence Related Secretion Systems, Fimbriae, and Toxins in O2:K1 *Escherichia coli* Isolated From Bovine Mastitis

**DOI:** 10.3389/fvets.2021.622725

**Published:** 2021-02-11

**Authors:** Min Sun, Xing Gao, Kejie Zhao, Jiale Ma, Huochun Yao, Zihao Pan

**Affiliations:** ^1^Institute of Veterinary Medicine, Jiangsu Academy of Agricultural Sciences, Nanjing, China; ^2^College of Veterinary Medicine, Nanjing Agricultural University, Nanjing, China; ^3^Key Lab of Animal Bacteriology, Ministry of Agriculture, Nanjing, China

**Keywords:** bovine mastitis, *Escherichia coli*, secretion system, type IV pilus, toxin

## Abstract

Mastitis remains a major infection of dairy cows and an important issue for the dairy farmers, and *Escherichia coli* (*E. coli*) bovine mastitis is a disease of significant economic importance in the dairy industry. Our study identified six isolates belong to phylogroup B2 from 69 bovine mastitis *E. coli* strains. Except for one serotype O1 strain, all group B2 isolates were identified into serotype O2 and showed significantly higher mortality in the mouse infection than other phylogroups' strains. Genomic analyses and further tests were performed to examine the role of secretion systems, fimbriae, and toxins during the systemic infection of O2:K1 strain BCE049. Two integral T6SS loci and three predicted effectors clusters were found to assemble the functional T6SS complex and deliver diverse toxic effectors to modulate bacterial virulence in the mouse infection model. A total of four T4SS loci were harbored in the BCE049 genome, three of them are encoded in different plasmids, respectively, whereas the last one locates within the bacterial chromosome at *FQU84_16715* to *FQU84_16760*, and was significantly involved in the bacterial pathogenicity. Numerous predicted pilus biosynthesis gene loci were found in the BCE049 genome, whereas most of them lost long fragments encoding key genes for the pili assembly. Unexpectedly, a type IV pilus gene locus locating at *FQU84_01405* to *FQU84_01335* in the plasmid 2, was found to be required for the full virulence of mastitis strain BCE049. It should be noted that a genetic neighborhood inserted with diverse genes is encoded by the plasmid 1, which harbors three prominent toxins including β-hemolysin, cytotoxic necrotizing factor 2 and cytolethal distending toxin type III. Consequent studies verified that these toxins significantly contributed to the bacterial pathogenicity. These findings provide a molecular blueprint for understanding the underlying mechanisms employed by the bovine mastitis *E. coli* to colonize in host and cause systemic infection.

## Introduction

Mastitis is an inflammation of the cow udder mostly triggered by the invasion of pathogenic bacteria. *Escherichia coli* (*E. coli*), one of the main pathogens involved in cases of bovine mastitis, is responsible for significant losses on the dairy farms (1–5). Currently, only a few distinctive features of the mastitis *E. coli* isolates have been unraveled. Generally, *E. coli* mastitis results in a subclinical and persistent pathology (6, 7), while in the extreme cases can lead to severe systemic clinical symptoms such as sepsis concurrent with fever (8, 9). The subclinical mastitis is mostly like an infection caused by the environmental opportunistic bacteria (10), but systemic infection may be a typical symptom of the extraintestinal pathogenic *E. coli* (ExPEC) disease. These two different infection phenotypes whether caused by different *E. coli* pathotypes should be further explored.

Numerous studies found that phylogroups A and B1 *E. coli* strains are the increased proportions of mastitis isolates (11), whereas these two phylogroups are traditionally associated with the commensal or intestinal pathogenic *E. coli* strains (12–14). Mammary gland is a sterile cavity under normal physiological conditions such as urinary tract and other extraintestinal organs and thus significantly different from the intestinal tract with diverse microbial communities naturally (15). Currently, most *E. coli* isolates from phylogroups B2 could cause severe inflammatory infection, sepsis with fever, and even sudden death and have been widely recognized as the highly virulent population of diverse ExPEC pathotypes (16). Although most ExPEC isolates from avian and swine origin also belong to phylogroups A and B1, numerous studies have confirmed that group B2 is their typical virulent population (17–19). Therefore, screening the highly virulent strains belonging to phylogroups D and B2 from the samples with acute systemic clinical symptoms but not subclinical phenotypes, just like have been done in many animal origin ExPECs (19, 20), may contribute to better understand the pathogenic mechanism of *E. coli* mastitis. Most recently, the group B2 strains have been reported to seem to have emerged in *E. coli* mastitis (21, 22) and shown high virulence with acute clinical symptoms, suggesting these strains should be identified as the typical mastitis associated with ExPEC for further study.

Although published data cannot identify the bovine mastitis *E. coli* as ExPEC directly, the mastitis isolates from phylogroups B2 should attract broad attention. Currently, secretion systems, fimbriae, toxins, O-antigens, and capsules have been identified as specific virulence factors in ExPEC from animal and human origins. To date, six pathways of effector secretion (types I–VI) have been described in *E. coli* (23, 24). Therefore, the type III secretion system (25), T4SS (26), and T6SS (27) were identified to deliver diverse effectors into the recipient *via* a needle injection process. Fimbriae or pili are short filamentous organelles used by bacteria to adhere to various host cell surfaces. In general, ExPEC strains carry a significantly higher number of fimbrial compared to fecal/commensal strains (28), including type 1, P, F1C, F9, and Auf fimbriae and type IV pili. Numerous toxins, including α-hemolysin, cytotoxic necrotizing factor 1 (CNF1), secreted autotransporter toxin (Sat), and protease involved in colonization (Pic) (29–33), have been identified to play the key roles during ExPEC infection. It should be noted that many necrotoxigenic *E. coli* (NTEC) strains isolated from newborn calves carry CNF2 and cytolethal distending (Cdt III) toxins (34), which may be also prevalent in the bovine mastitis *E. coli*.

In this study, we isolated 69 bovine mastitis *E. coli* strains from the milk of cows with mastitis and identified six isolates belong to phylogroup B2. Especially, the B2 strain BCE049 identified as serotype O2 showed higher mortality in the mouse infection than other strains. Thereby, we managed to identify the potential virulence factors from the secretion systems, fimbriae, and toxins for clarifying the strong pathogenicity of the O2 strain BCE049. Our study provides a molecular blueprint for understanding the underlying mechanisms employed by bovine mastitis *E. coli* to colonize in host and cause systemic infection.

## Materials and Methods

### Bacterial Isolation and Identification

More than 2,000 milk samples from the cows with clinical mastitis cases containing a hard or swollen udder or milk production decreased were submitted to our laboratory by the farm veterinarians from diverse provinces of China during the previous 4 years. The samples were coated on sheep's blood medium under aseptic conditions, cultured at 37°C for 24 h, and typical and dominant colonies were picked for further purification. The polymerase chain reaction (PCR) production of 16s RNA from the pure cultures was then sequenced to identify the bacterial species based on the >97% sequence identity using the NCBI Blast analysis. The morphology of the bacteria was observed under an optical microscope after conducting Gram staining.

### Bacterial Strains, Plasmids, and Growth Conditions

Bacterial strains and plasmids used for further study are listed in Supplementary Table 1. The *E. coli* strain BCE049 was isolated in our laboratory from the milk of a cow that was diagnosed with acute mastitis. For genetic manipulations, all strains were grown on LB medium at 37°C with aeration, supplemented with kanamycin (Kan, 50 μg/mL), ampicillin (Amp, 100 μg/mL), chloramphenicol (Clm, 25 μg/mL), nalidixic acid (Nal, 50 μg/mL), or 0.1 mM isopropyl-d-thiogalactopyranoside when necessary.

### Phylogenetic and Serotype Analyses in the *E. coli* Isolates

The phylogenetic analysis of *E. coli* isolates from the bovine mastitis was performed according to the updated multiplex PCR method, as the previously described (35). Mastitis-source isolates were generally divided into four phylogroups (A, B1, B2, and D). The identification of serotypes O1 and O2 in the mastitis *E. coli* isolates was performed using the specific PCR assays as described previously (36). The primers used in this study are listed in Supplementary Table 2.

### DNA Manipulations and Plasmid Construction

The creation, maintenance, and transformation of plasmid constructs followed standard molecular cloning procedures. DNA amplification, ligation, electroporation, and gel electrophoresis were carried out as described previously (37). All oligonucleotide primers are listed in Supplementary Table 2. Deletion mutants were constructed using λ red recombinase mutagenesis, as described previously (38). For complementation, the PCR fragments of target genes were cloned into the pGEN-Pcm, using *Nde*I and *Bam*HI restriction sites. For the periplasmic expression construct, target genes were amplified and cloned into pBAD/HisA to generate an N-terminal fusion with the PelB leader peptide and a C-terminal fusion with a His6 tag.

### Genome Sequencing, Assembly, Annotation, and Bioinformatics Analysis

Genomic DNA sample was isolated from the cell pellets with a Bacteria DNA Kit (OMEGA) according to the manufacturer's instructions. Genomic DNA was quantified by using TBS-380 fluorometer (Invitrogen). The library construction and sequencing were performed at Novogene Biotechnology Co., Ltd. (Beijing, China). Whole-genome sequencing was performed using a combination of PacBio RS and Illumina sequencing platforms. The Illumina data were used to evaluate the complexity of the genome and correct the PacBio long reads. First, we used ABySS (http://www.bcgsc.ca/platform/bioinfo/software/abyss) to do genome assembly with multiple-Kmer parameters and obtained the optimal results of the assembly (39). Second, canu (https://github.com/marbl/canu) was used to assemble the PacBio corrected long reads. Finally, GapCloser software was subsequently applied to fill up the remaining local inner gaps and correct the single-base polymorphism (https://sourceforge.net/projects/soapdenovo2/files/GapCloser/) for the final assembly results (40).

The *ab initio* prediction method was used to get gene models for strain BCE049. Gene models were identified using Glimmer3 (41). Then, all gene models were blast against non-redundant (NR in NCBI) database, SwissProt (http://uniprot.org), and COG (http://www.ncbi.nlm.nih.gov/COG) (42) to perform functional annotation by blastp module. In addition, tRNAs were identified using the tRNAscan-SE (v1.23, http://lowelab.ucsc.edu/tRNAscan-SE) (43), and rRNAs were determined using the RNAmmer (44) (v1.2, http://www.cbs.dtu.dk/services/RNAmmer/).

Phylogenetic analyses were performed following the procedures outlined by Bingle et al. (45). A ClustalW alignment was generated using the homologs of target protein (such as VgrG, Hcp or VirB4). A phylogenetic tree was constructed using the MEGA 7.0 with the neighbor-joining method with Poisson correction and 1,000 bootstrap replicates.

### Mouse Infection Assay

The mouse survival assay was carried out as described previously (46). Ten mice in each group were challenged by intraperitoneal injection with different strains at the designed doses and monitored for symptoms until 7 days postinfection. The negative-control group was challenged with an equal volume of sterile phosphate-buffered saline (PBS). To evaluate bacterial proliferation *in vivo*, the bacterial load assay was conducted. Five mice in each group were inoculated with 6 × 10^7^ colony-forming units (CFU) of different strains; blood was harvested, weighed, and homogenized in PBS at the designed time points postinfection. After that, the homogenized samples were serially diluted and plated on LB agar to enumerate the CFU.

### Growth Curves for T6SS Effectors' Toxicity Assays

The *E. coli* growth curves for T6SS effectors' toxicity assays were measured as described previously (47). Briefly, top 10 cells harboring expression plasmids were grown overnight in LB medium with shaking at 180 revolutions/min at 37°C and subinoculated at a starting OD 600nm of 0.05. Cultures were induced with 0.25% l-arabinose after 1.5 h of growth in LB. Cell growth was tracked by measuring the OD 600 every 1 h. The vector pBAD-hisA (Invitrogen) was used for production of cytoplasmic localization proteins, whereas the pBAD-pleB was used for production of periplasmic localization proteins. Periplasmic localization was achieved by fusion with a PelB leader sequence (48). The results represented the mean ± standard deviations (error bars) of three independent experiments.

### Cytotoxicity Assays

The cytotoxic effect of bacteria in HeLa cells was evaluated by lactate dehydrogenase (LDH) measurement using the CytoTox 96 non-radioactive cytotoxicity assay (Promega Corporation, USA) (49, 50). The percent cytotoxicity was calculated as [(sample OD_490_ – bacterial spontaneous OD_490_ – cell spontaneous OD_490_)/(cell maximum OD_490_ – cell spontaneous OD_490_)] × 100. LDH release was monitored every 20 min of incubation at a bacterium–cell ratio of 0.1 at 37°C.

### Adhesion Assays With HeLa Cells

Adhesion assays were performed as previously reported (51). The HeLa cells were cultured in 24-well cell plates and washed three times with PBS. The bacteria were suspended in Dulbecco modified eagle medium without antibiotics and fetal bovine serum to a density of 2 × 10^6^ CFU/mL. After infecting the cells in each well with 1 mL of the bacterial suspension, the plates were centrifuged at 800 × *g* for 15 min and incubated at 37°C for 90 min. Subsequently, the infected cells were washed five times and trypsinized or lysed to determine CFU. Serial dilutions of the cell lysate were plated onto LB agar, and the plates were incubated overnight at 37°C. Each assay was repeated independently three times.

### Statistical Analysis

Statistical analyses were performed using Prism 5.0 (GraphPad). A one-way analysis of variance (ANOVA) was used in the analysis of the cell adhesion, cytotoxicity, and bacterial growth results; a log-rank (Mantel–Cox) test was used in the analysis of the survival curves of infected mice, and a two-tailed Mann-Whitney *U* test was performed for the bacterial colonization in mouse infection model. Differences were defined as significant at *P* < 0.05 and indicated by ^*^ or ^**^.

## Results

### *E. coli* Was an Important Pathogen on Bovine Mastitis in China

**Figure 1:**
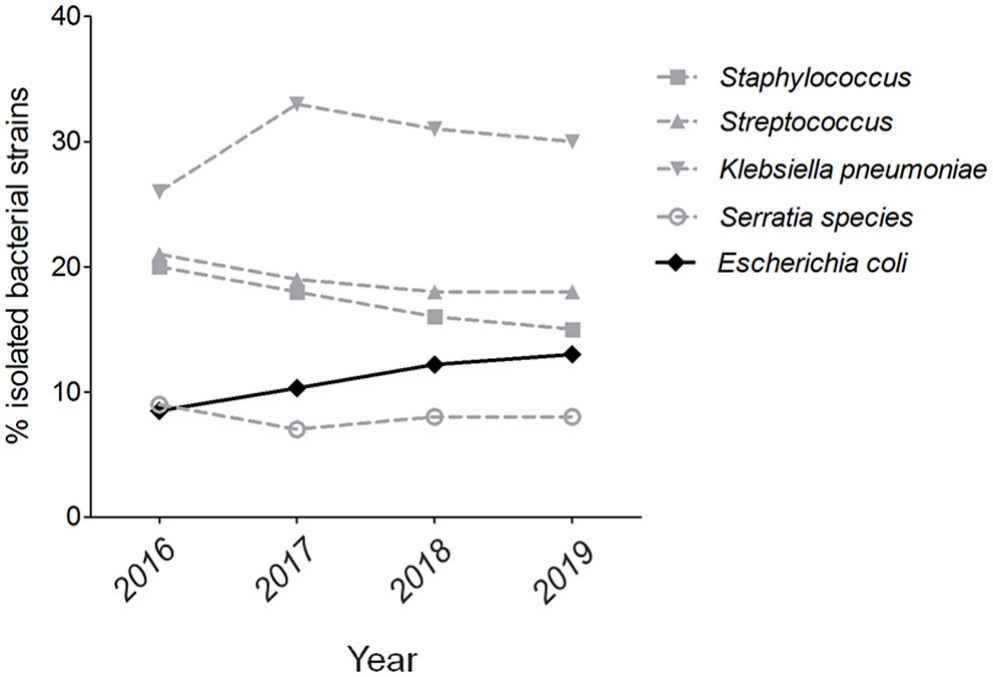
The isolation rate of *E. coli* strains on bovine mastitis in China. The isolation rates of five mainly pathogenic species in bovine mastitis from 2016 to 2019.

More than 2,000 milk samples were submitted to our laboratory by the farm veterinarians from diverse provinces of China during the previous 4 years. By the isolation of dominant bacterial colonies, a total of 627 strains were identified by 16s rRNA sequencing, including 232 gram-positive pathogenic, 301 gram-negative pathogenic, and 94 commensal bacteria. Therefore, 69 strains were identified as *E. coli*, showing a percentage at 11% in all isolated species and more than 20% of gram-negative pathogenic isolates. Further analysis of five mainly pathogenic species in bovine mastitis found that the isolation rate of *E. coli* shows a weak growth trend from 2016 to 2019 (Figure 1). These suggest that *E. coli* should be monitored constantly as an important pathogen on bovine mastitis.

### Serotype and Phylogenetic Analyses in the *E. coli* Isolates From Bovine Mastitis

**Figure 2:**
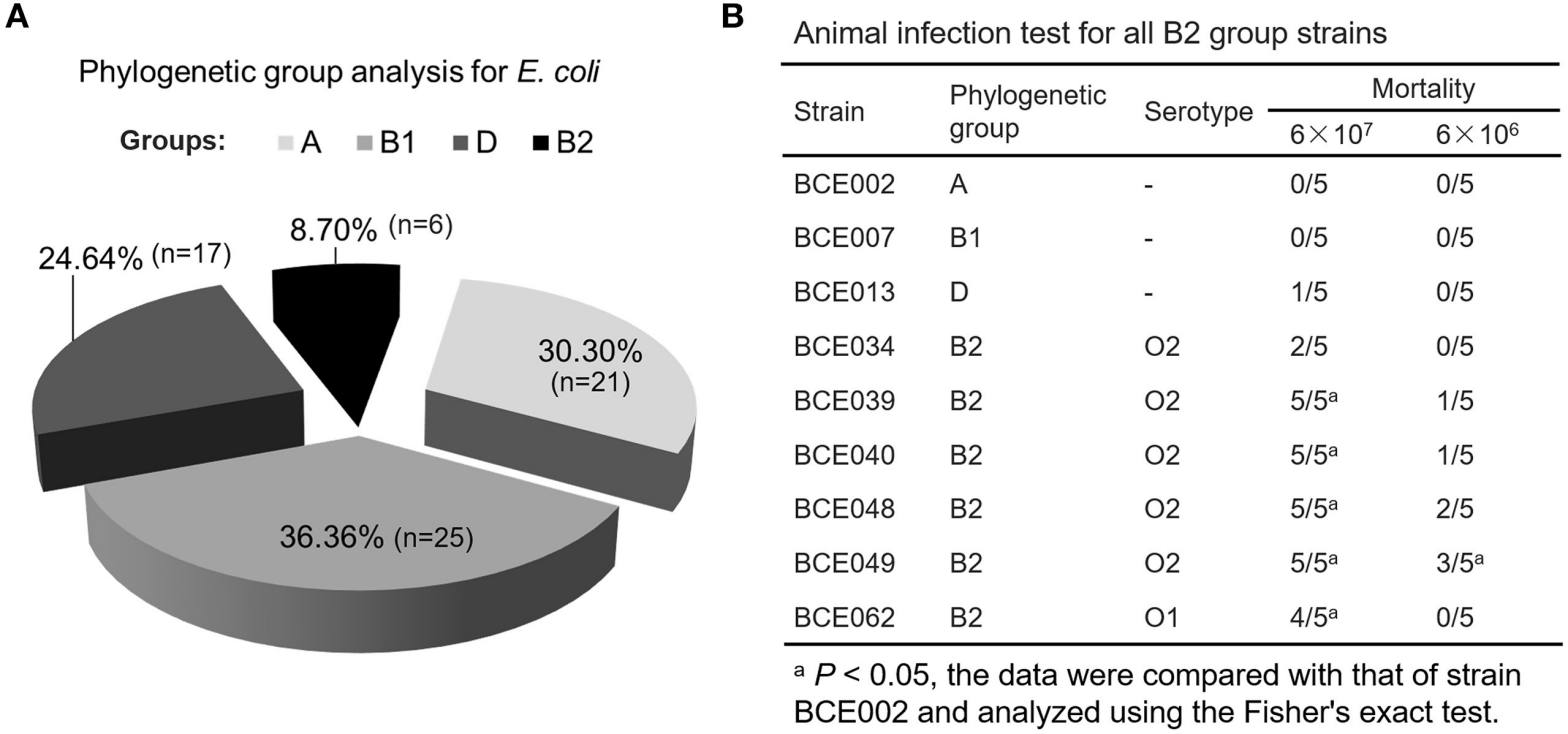
The pathogenicity of phylogenetic group B2 *E. coli* isolated from the bovine mastitis. **(A)** The proportion of *E. coli* isolates belonging to different phylogenetic groups. **(B)** Animal infection test for all B2 group strains.

Based on the pathogenic characteristics, the identified *E. coli* isolates from bovine mastitis were proposed to be classified as the extraintestinal pathogenic *E. coli*. To further assess their pathogenicity, the phylogenetic grouping was performed for preliminary analysis. As shown in Figure 2A, only 17 and 6 strains belong to groups D and B2, respectively. Some previous studies have reported that most of ExPEC strains from groups D and B2 showed higher pathogenicity than that of other two groups (16), which guide us to perform a further virulence test using a mouse infection model. As expected, no mortality was observed in mice challenged with group A strain BCE002 or group B1 strain BCE007 at the highest dose 6 × 10^7^ CFU after 7 days postinfection (Figure 2B). However, all group B2 strains showed significantly higher mortality in challenged mice than that of strains from the other three groups. Especially, the strain BCE049 showed the highest 100% and 60% mortality in mice challenged with 6 × 10^7^ CFU and 6 × 10^6^ CFU, respectively (Figure 2B). Using the serotype-specific PCR tests, almost all group B2 strains were identified as O2 serotype, except for the strain BCE062, which belongs to the O1 serotype (Figure 2B). Moreover, the genes encoding seven potential effectors of T6SS were detected using the PCR assay, which showed the different prevalence in the six B2 isolates identified by this study (Supplementary Table 3). These data indicate that these strains are not derived from the same clone. These results indicated that O2 is the dominant serotype in group B2 ExPEC isolated from mastitis in this study, and their pathogenic mechanism should be further explored.

### Whole-Genome Analysis Screened Potential Virulence-Related Secretion Systems, Fimbriae, and Toxins in O2:K1 Strain BCE049

**Figure 3:**
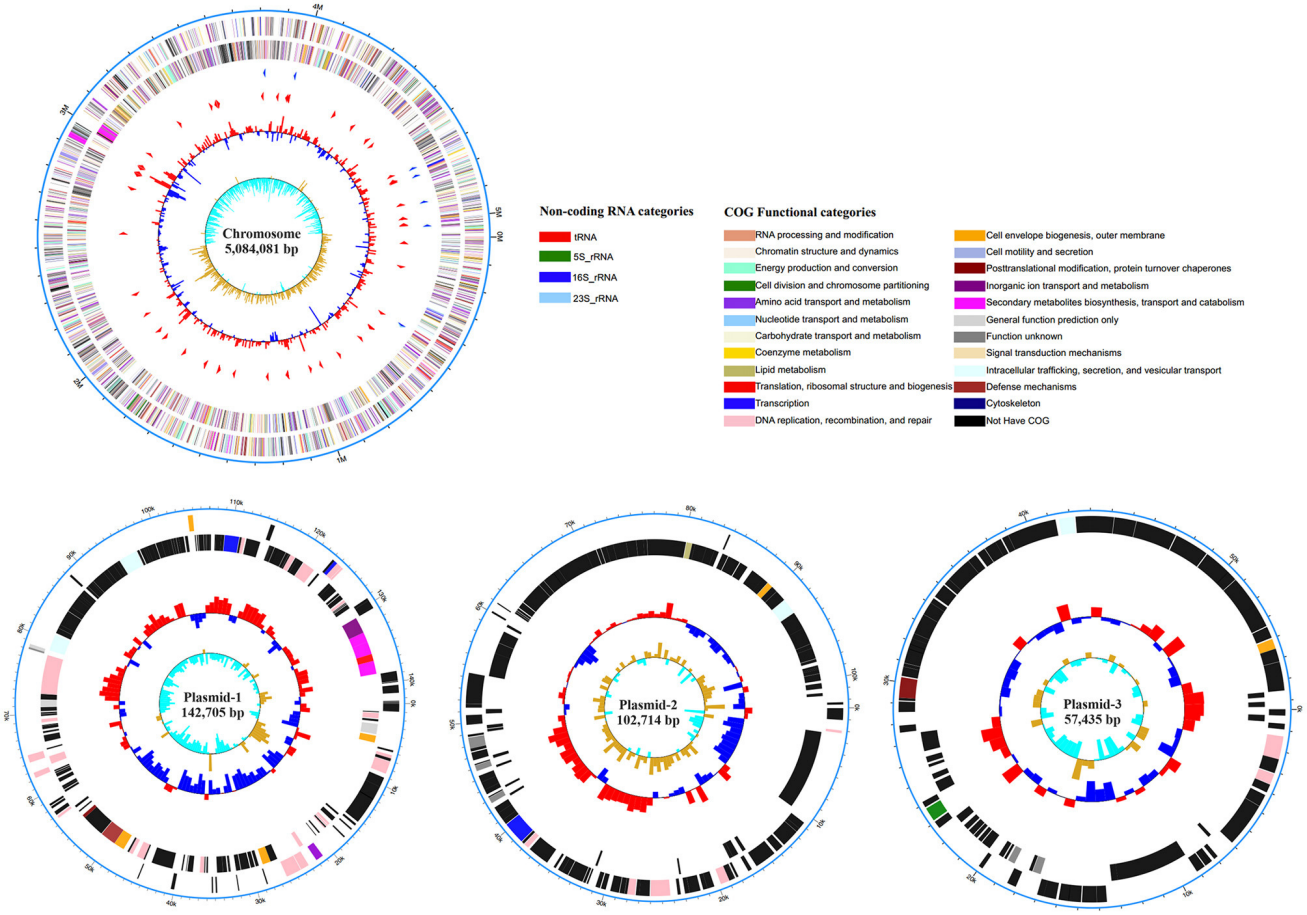
Circular representation of the chromosome and plasmids of strain BCE049 and related annotations. Maps were established using the software Circos v0.64 (http://circos.ca/). The key information pertains to the circular diagrams (outside to inside): the outermost rings show scales of the BCE049 chromosome and plasmids, where each division is 0.5 Mb and 10.0 kb, respectively; the second rings show the coding sequence (CDS) on the positive chains, and the different colors indicate the functional classification of different Clusters of Orthologous Group (COGs) of the CDS; the third rings show the CDS on the negative chains, and the different colors indicate the functional classification of different COGs of the CDS; the fourth ring shows non-coding genes (rRNA, tRNA, tmRNA); the innermost ring shows GC skew [(G – C)/(G + C)].

To gain insight into the possible genomic clues to the pathogenic mechanism of the virulent BCE049 strain, the complete genome was sequenced and submitted to GenBank database under the accession number CP042250, CP042249, CP042248, and CP042247 for chromosome and plasmids. The genome consisted of a single circular chromosome and three plasmids, approximately 5.08 Mb, 142.70 Kb, 102.71 Kb, and 57.43 Kb in sizes, respectively (Figure 3). The information of virulence factors in the pathogenic *E. coli* from bovine mastitis is scant currently; thus, the genomic analysis was performed by referring to other pathotypes of ExPEC. Previous studies have reported diverse secretion systems, fimbriae, and toxins that play important roles in the pathogenic process of ExPEC (16, 52). These virulence factors also were extensively encoded within the chromosome and plasmids of strain BCE049 and listed in Table 1 for further study here. Some genes that encoded the homologs of fimbriae or potential effectors of secretion systems were predicted and listed together.

**Table 1:** Putative secretion systems, fimbriae, and pathotoxins from O2:K1 *E. coli* strain BCE049.

**Bacterial factor**	**Locus tag**	**Cluster size**	**No. of proteins**	**Characteristic**
**Secretion system**				
T6SS1	*FQU84_ 06625-515*	29,775 bp	22	Conserved components and potential effectors
T6SS2	*FQU84_ 21500-405*	25,889 bp	21	Conserved components and potential effectors
T6SS effector locus1	*FQU84_13600-645*	9,168 bp	8	VgrG, PAAR proteins and potential effectors
T6SS effector locus2	*FQU84_03280-285*	1,670 bp	2	Hcp and potential HNH nucleases effector
T6SS effector locus3	*FQU84_22055-040*	2,767 bp	4	Hcp and potential pyocin_S nucleases effector
T4SS-genome	*FQU84_16715-760*	9,779 bp	11	Type IV secretion system
T4SS-plasmid1	*FQU84_00555-380*	32,141 bp	36	Type-F conjugative transfer system
T4SS-plasmid2	*FQU84_01320-1160*	29,980 bp	32	Conjugative transfer system
T4SS-plasmid3	*FQU84_01720-1805*	14,761 bp	17	Conjugative transfer system
T2SS1	*FQU84_03835-900*	12,731 bp	15	Type II secretion system
T2SS2	*FQU84_05670-720*	10,345 bp	11	Type II secretion system
**Fimbria**				
Type 1 fimbria 1	*FQU84_03355-385*	7,156 bp	7	Type 1 fimbria
Type 1 fimbria 2	*FQU84_23120-160*	8,758 bp	10	Type 1 fimbria
P fimbria	*FQU84_23465-500*	7,674 bp	8	Type P fimbria
T4Pb- plasmid2	*FQU84_01405-1335*	12,220 bp	15	Type IV pili B
**Pathotoxin**				
Hemolysin-plasmid1	*FQU84_00230-215*	7,162 bp	4	RTX toxin hemolysin
CNF2-plasmid1	*FQU84_00185*	2,913 bp	1	Cytotoxic necrotizing factor-2
CdtABC-plasmid1	*FQU84_00150-140*	2,143 bp	3	Cytolethal distending toxin type III

### Identification of Toxic Effectors Delivered by T6SSs to Modulate the Bacterial Virulence in Mouse Infection Model

**Figure 4:**
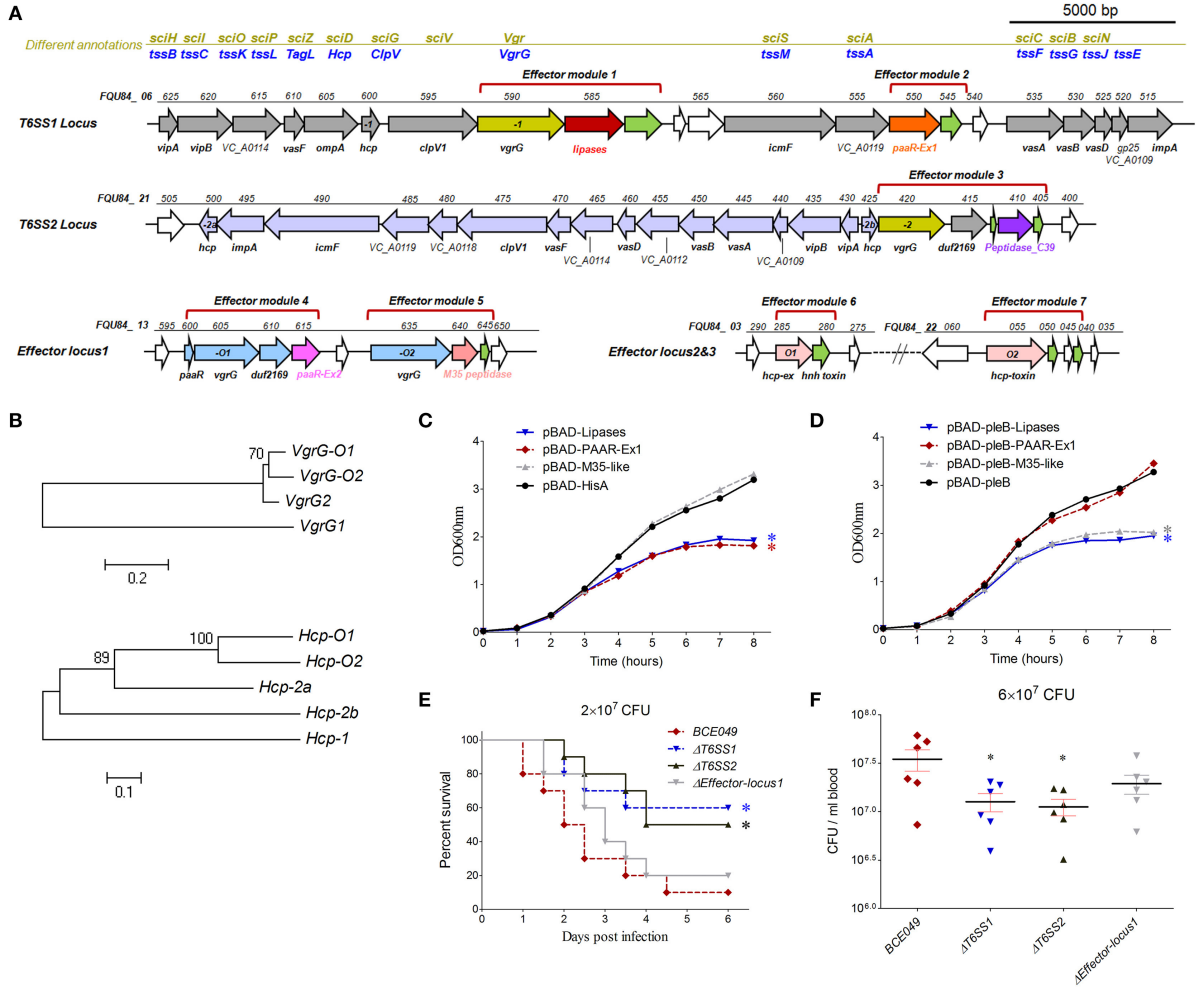
The role of T6SSs and predicted effectors in interbacterial competition and pathogenicity. **(A)** Schematic diagram of the genetic organization in two T6SS loci and three predicted effector loci. **(B)** The evolutionary relationships among VgrGs encoded within effector locus1 and T6SS loci in strain BCE049 (upper panel), and among Hcps encoded within effector locus2, locus3, and T6SS loci (lower panel). The protein sequences were aligned with ClustalW, and the MEGA software version 5.0 was used to perform a 1,000-bootstrap phylogenetic analysis using the neighbor joining method. **(C,D)** Growth curves of *E. coli* cells producing lipase, PAAR-Ex1 and M35-peptidase using pBAD-hisA or pBAD-pleB plasmids. The periplasmic localization of proteins using plasmid pBAD-pleB was achieved by fusion to the PelB leader sequence (48). The cultures were induced by l-arabinose at the indicated time (shown by the red arrow). The experiments were run in triplicate. The data were compared with that of strain with the vector pBAD-hisA or pBAD-pleB and analyzed using the one-way ANOVA test (*P < 0.05). **(E)** Effect of *hlyCABD* and *CNF2* on ExPEC BCE049 pathogenicity. Survival curve of mice infected with 2 × 10^7^ CFU/mouse bacteria (10 5-week-old mice per group). The data were compared with that of strain BCE049 and analyzed using the log-rank (Mantel–Cox) test (*P < 0.05). **(F)** Systemic infection experiments were conducted to assess bacterial proliferation in mouse blood. Bacterial reisolation from the blood at 12 h postinoculation was quantified by plate count. Statistical significance was determined by a Student *t*-test based on comparisons with the wild-type group (*P < 0.05).

Whole-genome search found two integral T6SS loci and three predicted effectors clusters encoded in the strain BCE049. Homology analysis of conserved proteins identified the first locus from *FQU84_ 06625* to *FQU84_ 06515* that encodes the T6SS1, and another locus from *FQU84_ 21500* to *FQU84_ 21405* that encodes the T6SS2 (Figure 4A). Further analysis for effectors prediction found two and one potential effector modules that encoded in the T6SS1 and T6SS2 loci, respectively. Three predicted effectors clusters locate at *FQU84_13600* to *FQU84_13645, FQU84_03280* to *FQU84_03285*, and *FQU84_22055* to *FQU84_22040*, which each encodes at least one potential effector module (Figure 4A). Taken together, a total of seven predicted effectors modules were found here and subsequently designated as modules 1 to 7 for further study. Therefore, modules 1, 3, 4, and 5 showed a close relationship with the VgrG protein for effectors secretion, whereas modules 6 and 7 encode extended Hcp proteins with C-terminal toxin fusion (Figure 4A). It is reasoned that the effectors from modules 1 and 2 may be secreted *via* T6SS1, and effector from module 3 may be secreted *via* T6SS2. To explore which T6SS mediated the secretion of effectors from modules 4–7, phylogenetic analyses for VgrG and Hcp proteins were performed. As shown in Figure 4B, the VgrG-O1 and -O2 from modules 4 and 5 showed closer relationship with the VgrG protein from T6SS2, suggesting their downstream effectors may be secreted *via* T6SS2 by the formation of VgrG heterotrimer. Similarly, the Hcp-O1 and -O2 from modules 6 and 7 showed closer relationship with the Hcp2a protein, suggesting their secretions may be mediated by the T6SS2 (Figure 4B).

Diverse T6SS effectors have been reported to associate with the interbacterial competition (53) and thus play a key role in bacterial survival and proliferation in the polymicrobial environments (54) and benefit pathogenic bacteria in invading new habitats in infected hosts. To verify whether our predicted effectors contributes to the interbacterial competition, the lipase from module 1, PAAR-Ex1 from module 2, and M35-pepetidase from module 5 were selected to construct the *E. coli* expression plasmids for testing the growth inhibition. The lipase and M35-pepetidase were predicted to damage the target cell membrane and wall, respectively. However, the reference information for PAAR-Ex1 predicted as periplasmic localization was unavailable. As shown in Figures 4C,D, a significant decrease in *E. coli* survival was shown when the periplasmic localized PAAR-Ex1 and M35-like were produced by the fusion of PleB leader sequence after l-arabinose induction, but not in the cytoplasmic localized productions. However, the growth cover assays showed that both the cytoplasmic and periplasmic lipase were toxic for the engineered *E. coli*. These results suggest that both T6SS1 and T6SS2 were functional in the effectors' secretion for interbacterial antagonisms.

We next managed to explore the potential pathogenic roles of these T6SSs in mastitis isolate BCE049. A mouse infection test was performed using BALB/c mice injected with 2 × 10^7^ CFU of T6SS1, T6SS2, and effector-locus1 deletion mutant and wild-type strains. As shown in Figure 4E, the mice infected with Δ*T6SS1* and Δ*T6SS2*, but not Δ*effector-locus1*, showed a significantly higher survival rate (>50%), compared with the survival of mice infected with the wild-type strain (10%). Further test of the bacterial loads in animal blood showed significant attenuations in the mice challenged with Δ*T6SS1* and Δ*T6SS2* compared to that of wild-type strain (Figure 4F). Altogether, our data indicated that both T6SS1 and T6SS2 were important for the full virulence of the mastitis ExPEC.

### The Chromosome-Encoding T4SS Was Involved in the Bacterial Pathogenicity

**Figure 5:**
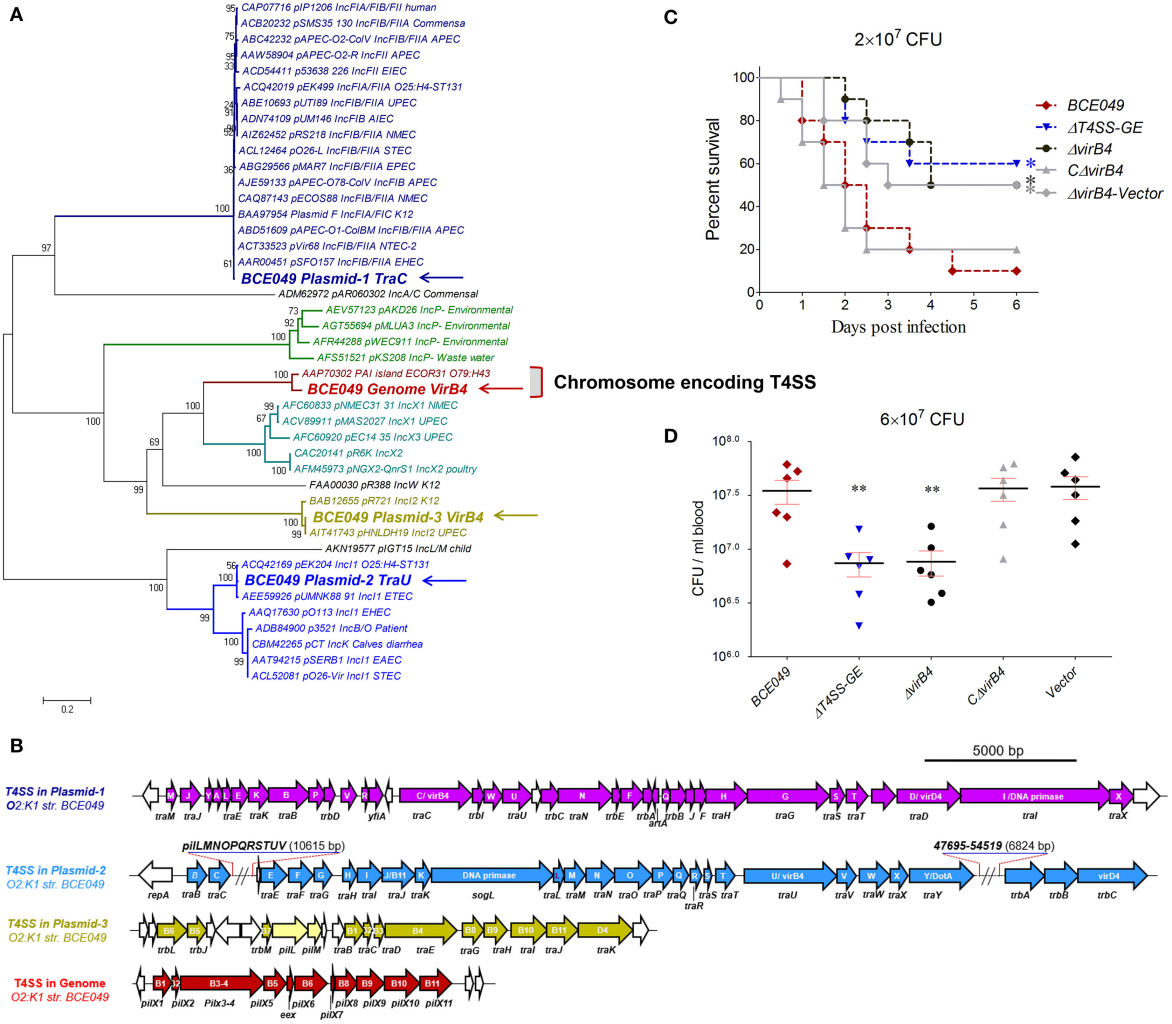
Identification of a chromosome-encoding T4SS contributing to bacterial virulence. **(A)** Phylogeny-based genotyping of 43 T4SSs with available VirB4 homolog amino acid sequences from various bacterial strains. A neighbor-joining tree (bootstrap *n* = 1,000; Poisson correction) was constructed based on a ClustalW alignment of the VirB4 amino acid sequences encoded in T4SS loci from diverse *E. coli* pathotype strains. **(B)** The genetic organizations of four T4SS loci from strain BCE049. **(C)** Effect of T4SS-CE and VirB4 on ExPEC BCE049 pathogenicity. Survival curve of mice infected with 2 × 10^7^ CFU/mouse bacteria (10 5-week-old mice per group). The data were compared with that of strain BCE049 and analyzed using the log-rank (Mantel–Cox) test (*P < 0.05). **(D)** Systemic infection experiments were conducted to assess bacterial proliferation in mouse blood as the description of Figure 4F legend (**P < 0.01).

It should be noted that a total of four T4SS loci were harbored in the mastitis isolate BCE049. Three of them are encoded in different plasmids, respectively, whereas the last one locates within the bacterial chromosome at *FQU84_16715* to *FQU84_16760*. The most common role of the plasmid-encoding T4SS is to mediate the conjugation of plasmid DNA (55); as a result, these systems contribute to the spread of exogenous DNA fragments (such as the plasmid-borne antibiotic resistance genes). A phylogenetic analysis of conserved VirB4 homologs showed that these four T4SSs were divided into three different deep clades (Figure 5A). Especially, the protein from chromosome-encoding T4SS (T4SS-CE) constituted an independent branch with the homolog encoded in pathogenicity island (PAI) of strain ECOR31 O79:H43, and both of them showed potential relationship with *E. coli* pathogenicity in the bioinformatics analysis (56). The genetic organization of chromosome-encoding T4SS locus showed a significantly shorter sequence containing only 11 conserved genes compared with the other three T4SSs (Figure 5B). Although more evidences about the pathogenic roles of this type T4SS are unavailable by searching for previous publications, we hypothesized that this T4SS might contribute to the bacterial virulence. Indeed, the mice infected by the Δ*T4SS-CE* and Δ*virB4* showed a significantly higher survival rate (>50%), compared with the 10% in wild-type challenged mice (Figure 5C). Moreover, the deletion of T4SS-CE or *virB4* also significantly attenuated the bacterial loads in mice blood compared with the wild-type strain (Figure 5D). Certainly, the above deficiencies of *virB4* deletion mutants were completely restored by complementation. These observations suggest that chromosome-encoding T4SS is a new virulence factor in mastitis ExPEC.

### The Roles of Type IV Pilus (T4P) Gene Cluster Encoded in the Plasmid 2 During the BCE049 Infection

**Figure 6:**
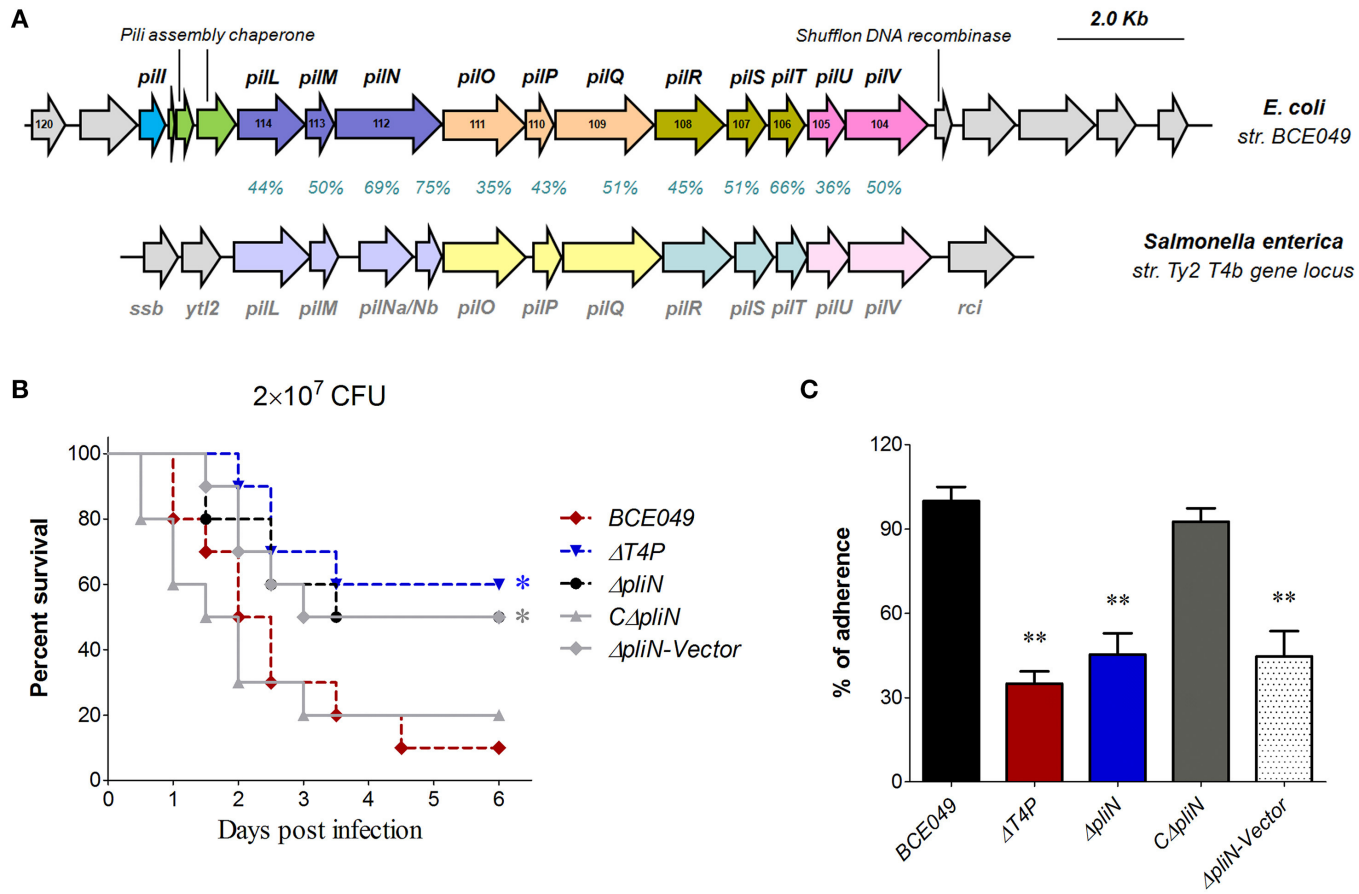
Identification of a plasmid-encoding T4P involved in bacterial virulence. **(A)** Schematic diagram for the genetic organization of T4P loci from *E. coli* strain BCE049 and *Salmonella enterica* strain Ty2. The identity and similarity of amino acid sequences for each gene in T4P loci are shown. **(B)** Effect of T4P and PliN on ExPEC BCE049 pathogenicity. Survival curve of mice infected with 2 × 10^7^ CFU/mouse bacteria (10 5-week-old mice per group). The data were compared with that of strain BCE049 and analyzed using the log-rank (Mantel–Cox) test (*P < 0.05). **(C)** HeLa cells adhesion assay. All assays were run in triplicate. Statistical significance was determined by Student *t*-test (**P < 0.01).

Numerous gene loci predicted for the pilus biosynthesis were found in the BCE049 genome (Table 1), whereas most of them lost long fragments encoding key genes for pili assembly. Only one integral pilus gene locus named type IV pilus (T4P), reported many times as the virulence factor in *Salmonella enterica, Pseudomonas aeruginosa*, and *Vibrio cholerae* previously (57–59), locates at *FQU84_01405* to *FQU84_01335* in the BCE049 plasmid 2. Further analysis showed that this T4P gene locus shares the highest sequence identity and similar gene encoding order with the homolog of *S. enterica* strain Ty2 (Figure 6A), suggesting it may also function in the bacterial pathogenicity. Therefore, the gene *pliN* encodes the core component played as the outer membrane protein of the type IVB pilus. A mouse infection test was performed using the BALB/c mice injected with 2 × 10^7^ CFU of related deletion mutants and wild-type strains. As depicted in Figure 6B, the mice infected by Δ*T4P* and Δ*pliN* showed a significantly higher survival rate (>50%), compared with the 10% in wild-type challenged mice, and the complementation in Δ*pliN* restored the above deficiency. It is reasoned that many pilus structures play key roles in bacterial adherence during the early infection. Indeed, the adherences of Δ*T4P* and Δ*pliN* were significantly reduced in HeLa cells compared with that of the wild-type strain (Figure 6C). Altogether, our data indicated that this T4P is important for the full virulence of the strain BCE049.

### The Contributions of the Plasmid-Encoding CNF2 and Hemolysin (HlyCABD) in the Bacterial Pathogenicity

**Figure 7:**
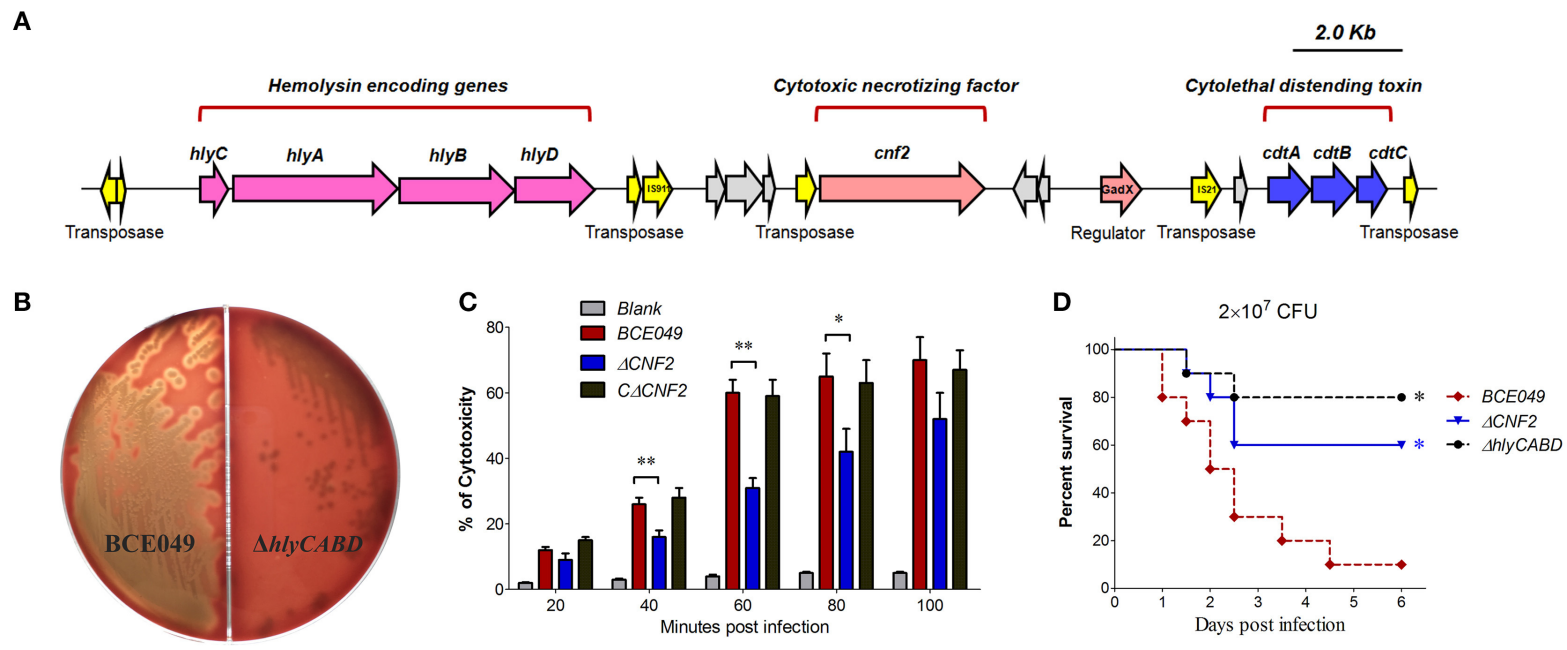
The plasmid-encoding toxins are required for the full virulence of strain BCE049. **(A)** Schematic diagram of the genetic organization. **(B)** Hemolysis test on sheep blood plate cultured 20 h at 37°C. **(C)** Assessment of the cytotoxicity in HeLa cells. An MOI of 0.1 bacterium/cell (2 × 10^4^ CFU bacteria/well) was chosen to study the kinetics of cytotoxicity by *E. coli* strains. **(D)** Effect of *hlyCABD* and *CNF2* on ExPEC BCE049 pathogenicity. Survival curve of mice infected with 2 × 10^7^ CFU/mouse bacteria (ten 5-week-old mice per group). The data were compared with that of strain BCE049 and analyzed using the log-rank (Mantel–Cox) test (*P < 0.05; **P < 0.01).

It is well-known that CNF and hemolysin play key roles in bacterial pathogenicity (52) and were encoded in chromosome of diverse *E. coli* pathotypes. In this study, we found a genetic neighborhood with diverse genes insertion from the plasmid 1, which harbored three toxin loci including the *hlyCABD, CNF2*, and *cdtABC* (Figure 7A). To verify whether they play similar roles with the chromosome-encoding homologs, their toxicity tests were performed by constructing different deletion mutants. As shown in Figure 7B, the deletion of *hlyCABD* caused the disability of β-hemolysis when bacteria were cultured on the sheep blood plates, but not in the wild-type strain. Subsequently, a multiplicity of infection was chosen to study the CNF2 cytotoxicity of HeLa in related deletion mutants and wild-type strains. The inactivation of CNF2 caused the significant attenuation of cell damage at 40 to 80 min postinfection (Figure 7C), suggesting that strain BCE049 was able to kill the host endothelial cells *via* the CNF2 toxin due to the bacterial colonization. To further test the functions of plasmid-encoding CNF2 and hemolysin, animal infection tests were performed. As expected, the mice infected by theΔ*CNF2* and Δ*hlyCABD* showed a significantly higher survival rate (>60%), compared with the 10% in wild-type challenged mice (Figure 7D). These observations indicated that plasmid-encoding CNF2 and hemolysin also are important virulence factors in the mastitis ExPEC.

## Discussion

The aim of the present report was to identify the important virulence factors for animal systemic infection in the emerging phylogroup B2 *E. coli* isolated from bovine mastitis. Except for one serotype O1 strain, all group B2 isolates were identified into serotype O2 and showed significantly higher mortality in the mouse infection than other phylogroups' strains. Genomic analyses of O2:K1 strain BCE049 found four T6SS-related, one T4SS-related, two type 1 fimbria–like, and one type P fimbria–like loci are encoded within the chromosome, and three T4SS-related, one type IV pili, one hemolysin A–like, one CNF2–like, and one cytolethal distending toxin type III–like loci are encoded within the plasmid 1, 2, or 3. Our present study demonstrated that T6SS1, T6SS2, chromosome-encoding T4SS, type IV pili, hemolysin A, and CNF2 are required for the full virulence of strain BCE049 in the mouse infection model.

Among the secretion systems, T4SSs have the unique ability to mediate the translocation of DNA (in addition to proteins) into bacterial or eukaryotic target cells (55). Their most common role is to mediate the conjugation of plasmid DNA, thus contributing to the spread of plasmid-borne antibiotic resistance genes. Although T4SSs are involved in the bacterial pathogenesis by translocation of protein effectors in a few organisms (55), this is never reported in *E. coli* and remains unclear. Expectedly, three plasmid-encoding T4SSs in strain BCE049 were identified as the homologs for DNA conjugation from plasmids IncF, IncI2, and IncI3 (60, 61), respectively. However, a chromosome-encoding T4SS was found in this study and showed a close relationship with the homolog encoded in PAI of strain ECOR31 O79:H43. Especially, the latter has been reported as the putative progenitor of the *Yersinia* high-pathogenicity island by mediating virulence genes' integration into the genome (56), suggesting this type T4SS has the potential relevance with the bacterial pathogenicity. Although there is no evidence of virulence factors' secretion *via* this chromosome-encoding T4SS, our data showed that its deletion significantly attenuates the bacterial virulence and loads in host blood compared to the wild-type strain. Moreover, this type T4SS locus was retrieved against the NCBI database, which showed that the encoding sequence was widely distributed in the chromosomes of numerous *Klebsiella pneumoniae* and several multidrug-resistant *E. coli* strains. This finding suggests that the chromosome-encoding T4SS locus may be acquired by the strain BCE049 from the close relative species *K. pneumoniae* of Enterobacteriaceae *via* the horizontal gene transfer.

T4P are surface-exposed protein fibers that perform a variety of functions in the bacterial life cycle, including twitching motility, adhesion, biofilm formation, and horizontal genetic transfer (62), and are classified as T4aP or T4bP based on the differences in the components of assembly system. The chromosome encoding T4P is more diverse and best characterized for enteric bacteria such as enteropathogenic, enterohemorrhagic, and enterotoxigenic *E. coli, S. enterica* serovar *typhi*, and *V. cholerae* (63). In contrast to the established functions in intestinal pathogenic *E. coli*, the roles for T4P in the virulence of ExPEC have not been well-established (64). In this study, we found a plasmid encoding T4P locus in the mastitis isolate BCE049, which shares the highest sequence identity and similar gene encoding order with the homolog of *S. enterica* strain Ty2 and thus is classified as a homolog of T4bP. It should be noted that T4aP aids in dispersal across various surfaces *via* the flagella-independent twitching motility, whereas T4bP is usually involved in the adherence and aggregation (65). Our data confirmed that this plasmid encoding T4bP really contributes to the optimal adherence to host epithelial cell in the mastitis *E. coli*. The prevalence and pathogenic roles of plasmid encoding T4P need be extensively concerned and further explored.

NTEC is a group of pathogenic *E. coli* strains defined by the production of CNF1, CNF2, or CNF3 variants found in diverse hosts (66–68). The gene locus for hemolysin was reported to show the strongest correlation to *cnf1* in uropathogenic *E. coli* (UPEC) (30). Numerous UPEC isolates positive for *cnf1* had the β-hemolysin genes *hlyCABD* immediately upstream, thus forming the hemolysin-*cnf1* operon (69). Different from the above chromosome encoding hemolysin-*cnf1* operon, mastitis strain BCE049 was found to encode a hemolysin-*cnf2* operon in plasmid. Although its upstream four genes showed a high sequence identity with the well-known hemolysin genes *hlyCABD* and really function for β-hemolysin, the downstream *cnf2* gene has never been found in the human ExPEC. It should be noted that *cnf2* gene was extensively encoded in the strains isolated from newborn calves with the significant extraintestinal infection (34) and significantly involved in the bacterial virulence, suggesting this plasmid encoding hemolysin-*cnf2* operon may be closely related to the bovine infection, including cows and calves. In fact, similar plasmid harbored by *E. coli* isolates from the septicemic calves and lambs (34, 70) was designated as Vir plasmid, which was reported to propagate the *cnf2* and *f17A* genes in NTEC2 cattle isolates (71) and other toxic genes, such as *cdtIII* (72). Taken together, its pathogenic roles in mastitis *E. coli* are worthy of further exploration.

The definition of a mastitis-associated pathotype, along with criteria specific for these strains, is the cornerstone of following studies in bovine mastitis *E. coli*. So far, multiple studies have failed to clarify a specific virulence determinants' set associated with the mastitis strains (73–76). One of the probable reasons may be that too many commensal and intestinal *E. coli* strains causing subclinical phenotypes have been mixed with the highly virulent strains. In fact, similar difficulties commonly exist in the isolation of ExPEC from animal origin, but not in that of human uropathogenic and neonatal meningitis *E. coli* (UPEC and NMEC) (19). It is easy to imagine that the breeding environments of most economic animals contain more pollutions with feces, sewage, and bacteria comparing with the daily living surroundings of human. In this study, given the acute bacteremia and mastitis caused by the O2:K1 strain BCE049, we classified this phylogroup B2 isolate into the ExPEC population. Although some previous studies have established effective mastitis model in mouse to assess the virulence of bovine mastitis *E. coli* (77), our results suggest that the high virulence of the group B2 strains could not be verified using the mastitis model, which may be better represented after the bacterial cells breaking the host blood barrier using the systemic infection model.

In summary, our study comprehensively examined the role of secretion systems, fimbriae, and toxins during the systemic infection of phylogroup B2 *E. coli* isolated from bovine mastitis. These findings provide the compelling evidence that the effective assembly of T6SS1, T6SS2, chromosome-encoding T4SS, and type IV pili and the export of hemolysin A and CNF2 toxins are required for the bacterial full virulence in the mastitis strain BCE049, which contributed to a better understanding of the pathogenic mechanism of bovine mastitis *E. coli*. These virulence factors may serve as therapeutic targets for counteracting or preventing the colonization of phylogroup B2 *E. coli* in the clinical bovine mastitis.

## Data Availability Statement

The datasets presented in this study can be found in online repositories. The names of the repository/repositories and accession number(s) can be found in https://www.ncbi.nlm.nih.gov/genbank/, CP042250; https://www.ncbi.nlm.nih.gov/genbank/, CP042249; https://www.ncbi.nlm.nih.gov/genbank/, CP042248; https://www.ncbi.nlm.nih.gov/genbank/, CP042247.

## Ethics Statement

The animal study was reviewed and approved by Ethics Committee for Animal Experimentation of Nanjing Agricultural University.

## Author Contributions

MS and ZP contributed by preparing the main text of the manuscript. MS is responsible for all the planning, experimenting, and analysis of the data presented in this paper. MS, XG, KZ, JM, and ZP performed the experiments. ZP and HY are responsible for supervising the study and reviewing the manuscript. All authors have read and agreed to the published version of the manuscript.

## Conflict of Interest

The authors declare that the research was conducted in the absence of any commercial or financial relationships that could be construed as a potential conflict of interest.
