# Supplementary material for: Insight Into the Virulence Related Secretion Systems, Fimbriae, and Toxins in O2:K1 Escherichia coli Isolated From Bovine Mastitis
Source: Front Vet Sci. 2021 Feb 11;8:622725. doi: 10.3389/fvets.2021.622725 (PMC7904677; doi:10.3389/fvets.2021.622725)
Supplement: Supplementary file 1 [file Table_1.DOC]

**Table S1 Summary of bacterial strains and plasmids for the study of potential virulent factors.**

| Strain or plasmid | Characteristic(s) | | Source |
| --- | --- | --- | --- |
| **Strains** | | | |
| BCE049 | | Virulent O2:K1 *E. coli* strain isolated from bovine mastitis | Clinical isolation |
| △*T6SS1* | | Deletion mutant of T6SS1 locus with BCE049 background | This study |
| △*T6SS2* | | Deletion mutant of T6SS2 locus with BCE049 background | This study |
| △*Effector locus1* | | Deletion mutant of T6SS effector locus1 with BCE049 background | This study |
| △*T4SS-GE* | | Deletion mutant of genome encoding T4SS with BCE049 background | This study |
| △*virB4* | | Deletion mutant of *virB4* of genome encoding T4SS with BCE049 background | This study |
| △*T4P* | | Deletion mutant of Type IV pili locus with BCE049 background | This study |
| △*pliN* | | Deletion mutant of *pliN* of Type IV pili locus with BCE049 background | This study |
| △*CNF2* | | Deletion mutant of *CNF2* with BCE049 background | This study |
| △*hlyCABD* | | Deletion mutant of hemolysin locus *hlyCABD* with BCE049 background | This study |
| C△*virB4* | | BCE049 △virB4 with the vector pGEN-virB4 | This study |
| C△*pliN* | | BCE049 △pliN with the vector pGEN-pliN | This study |
| C△*CNF2* | | BCE049 △CNF2 with the vector pGEN-CNF2 | This study |
| *E. coli* Top10 | | Cloning host for maintaining the recombinant plasmids | [Solarbio](http://www.baidu.com/link?url=VSoWIkrcO8ir_XFJZ_HVfhu4SpcRd4kSSjphZKP6TDsWU3k9OMyXlChSn6gBKNah) |
| **Plasmids** | |  |  |
| pBAD/HisA | | Expression vector, Amp^R^ | Invitrogen |
| pGEN-*pcm* | | *E. coli* shuttle vector pGEN MCS with a Pcm promoter; Amp^R^ | Laboratory stock |
| pBAD-*pleB* | | pBAD/HisA with a leader sequence encoding PleB for [fusion](../../../../C:%5CUsers%5Cjialema%5CAppData%5CLocal%5Cyoudao%5Cdict%5CApplication%5C7.5.0.0%5Cresultui%5Cdict%5C%3Fkeyword=fusion)[expression](../../../../C:%5CUsers%5Cjialema%5CAppData%5CLocal%5Cyoudao%5Cdict%5CApplication%5C7.5.0.0%5Cresultui%5Cdict%5C%3Fkeyword=expression) | Laboratory stock |
| pGEN-virB4 | | pGEN MCS carrying *virB4* under the control of Pcm promoter | This study |
| pGEN-pliN | | pGEN MCS carrying *pliN* under the control of Pcm promoter | This study |
| pGEN-CNF2 | | pGEN MCS carrying *CNF2* under the control of Pcm promoter | This study |
| pBAD-Lipases | | pBAD-HisA carrying Lipases coding region | This study |
| pBAD-PAAR-Ex1 | | pBAD-HisA carrying PAAR-Ex1 coding region | This study |
| pBAD-M35-like | | pBAD-HisA carrying M35-like coding region | This study |
| pBAD-pleB-Lipases | | pBAD-*pleB* carrying Lipases coding region | This study |
| pBAD-pleB-PAAR-Ex1 | | pBAD-*pleB* carrying PAAR-Ex1 coding region | This study |
| pBAD-pleB-M35-like | | pBAD-*pleB* carrying M35-like coding region | This study |
| pKD46 | | Red recombinase expression plasmid |  |
| PKD4 | | pANTS derivative containing FRT-flanked kanamycin resistance |  |
| pCP20 | | TS replication and thermal induction of FLP synthesis |  |

Datsenko, K. A., & Wanner, B. L. (2000). One-step inactivation of chromosomal genes in Escherichia coli K-12 using PCR products*. Proc Natl Acad Sci U S A,* 97(12), 6640-6645. doi:10.1073/pnas.120163297
