# Supplementary material for: Insight Into the Virulence Related Secretion Systems, Fimbriae, and Toxins in O2:K1 Escherichia coli Isolated From Bovine Mastitis
Source: Front Vet Sci. 2021 Feb 11;8:622725. doi: 10.3389/fvets.2021.622725 (PMC7904677; doi:10.3389/fvets.2021.622725)
Supplement: Supplementary file 2 [file Table_2.DOCX]

**Table S2. Primers used for PCR amplification.**

| Primer | Sequence(5'-3') |
| --- | --- |
| ***General PCR for cloning*** | |
| *PAAR-Ex1*-F | GGCGGATCCATGGGAACAGGCTACTTTCTG |
| *PAAR-Ex1*-R | GTGTCTAGATTATTGAACCCACTCCTCAGG |
| *M35-like*-F | GGCGGATCCATGGACGATAATAATCAAACT |
| *M35-like*-R | GTGTCTAGATTAATGGAGAAGTGCTTTTTC |
| *virB4*-F | ATCCATATGAAGCTGCTCAAGGCCATGAAA |
| *virB4*-R | CTCGGATCCTCAGAGCGCTTTAGCCAGGTA |
| *pliN*-F | ATCCATATGAAAAAATCACACCAGCGT |
| *pliN*-R | CTCGGATCCTCAGCCATTATTGCCTCCTGACGG |
| *CNF2*-F | ATCCATATGCAATGGCAACAAAAATATCTT |
| *CNF2*-R | CTCGGATCCTCATTTTGAAAAAACATGCAATAC |
| ***For Deletion^a^*** | |
| Del-T6SS1-F | TCACTCATCGGCGTCTTCCCCATACTCTTCCGCATCCTTCATACTCCTGCgtgtaggctggagctgcttc |
| Del-T6SS1-R | CAGTATGGGTTAAGGCGACGCAGCAGCGCGGCGGGGTGATTTCCTGTCATatgggaattagccatggtcc |
| Del-T6SS2-F | ATGATCCAGATTGATTTAGCCACGCTCGTAAAGCGGCTTAACCCCTTTGCgtgtaggctggagctgcttc |
| Del-T6SS2-R | TCACCGGCGGTCTGTTCAGCCTGTTCCGTTTACCGGATACGTTGTATTAAatgggaattagccatggtcc |
| Del-Effector locus1-F | ATGCCTTTAGCAGCCAAACTTACCGACAAAGGCACCCAGCACGACGGTTAgtgtaggctggagctgcttc |
| Del-Effector locus1-R | AGTTATGGGCAATTGAGGATGAAAAAAAATTCTATTTACGTCACCCCTGAatgggaattagccatggtcc |
| Del- *T4SS-CE*-F | ATGAATATGTTTAACAGGCTAAAGAGCGCCATCCCCCATGTTTTACCTTCgtgtaggctggagctgcttc |
| Del-*T4SS-CE*-R | ATTTCCGCCCGGCCCATCGCAATCAATATTTTGAGGAGATGAAAGCATGAatgggaattagccatggtcc |
| Del- *virB4*-F | ATGGCAAAGCTGCTCAAGGCCATGAAACGCCCGGCAGCGCTGTGGGGGGTgtgtaggctggagctgcttc |
| Del- *virB4*-R | GAATGCGGCCTCATGAATGGCTCGATACCTACCTGGCTAAAGCGCTCTGAatgggaattagccatggtcc |
| Del- *T4P*-F | TTAATTGAGCGTTACACACGACGCAATACAACCTGTCTTATGCTCGTATTgtgtaggctggagctgcttc |
| Del-*T4P*-R | TGTGCGTCGACGACCAGTATTTGCAGTCGTCCGGGATGTTGCTGTGGCATatgggaattagccatggtcc |
| Del- *pliN*-F | TCAGCCATTATTGCCTCCTGACGGGAAAGTCGGGGTAATGATGATCACAAgtgtaggctggagctgcttc |
| Del-*pliN*-R | ATGAAAAAATCACACCAGCGTTCAATGAAGCTGGCGGTGCTCCCCTGCATatgggaattagccatggtcc |
| Del-*CNF2*-F | TCAAAAATCTTTTGAAAAAACATGCAATACTGATATATTGGAGTTTTCTAgtgtaggctggagctgcttc |
| Del-*CNF2*-R | ACCAACTCATTGTACTCAAGAAGATATTTTTGTTGCCATTGAACGTTCATatgggaattagccatggtcc |
| Del- *hlyCABD*-F | TTAACGCTCATGTAAACTTTCTGTTACAGACTCTTCCAGAGGACTAAGAAgtgtaggctggagctgcttc |
| Del-*hlyCABD*-R | CTGGCCCAGAGCCAAGATACATGCCCAAGAACCTCTAATGGATTGTTCATatgggaattagccatggtcc |
| ***Primers for detection*** | |
| *E coli*-O1-F | GTGAGCAAAAGTGAAATAAGGAACG |
| *E coli*-O1-R | CGCTGATACGAATACCATCCTAC |
| *E coli*-O2-F | AGTGAGTTACTTTTTAGCGATGGAC |
| *E coli*-O2-R | AGTTTAGTATGCCCCTGACTTTGAA |
| *chuA-*F | GACGAACCAACGGTCAGGAT |
| *chuA-*R | TGCCGCCAGTACCAAAGACA |
| *yjaA-*F | TGAAGTGTCAGGAGACGCTG |
| *yjaA-*R | ATGGAGAATGCGTTCCTCAAC |
| *TspE4C2-*F | GAGTAATGTCGGGGCATTCA |
| *TspE4C2-*R | CGCGCCAACAAAGTATTACG |
| T6-Ex1-F | CGTGAGAAACCTGTACAGCCA |
| T6-Ex1-R | ATTGGTGGATGAGAATCTATT |
| T6-Ex2-F | GCGTTTTGATTTCTCATCATA |
| T6-Ex2-R | TTGTGGATTAGAACAATTACA |
| T6-Ex3-F | AAGTACCCAATGCCCTACTTT |
| T6-Ex3-R | TTTGGAGAGAACACACTTACT |
| T6-Ex4-F | GCTGGAGAAGAACGCAACGCT |
| T6-Ex4-R | TCACCCTGCTGCTCATACATC |
| T6-Ex5-F | GTGAAAGAGCAAAAAATAACA |
| T6-Ex5-R | ATCCCCATAATACCACTGAAT |
| T6-Ex6-F | TTATGAACAACTGTCCATGAT |
| T6-Ex6-R | TTAAAAAAAATGATGAATGAT |
| T6-Ex7-F | CACTTGAAAACTGCGCTATCA |
| T6-Ex7-R | GGTCTATGCCAAATCCTGTCT |

Underlined showed restriction cutting sites
