# Supplementary material for: Insight Into the Virulence Related Secretion Systems, Fimbriae, and Toxins in O2:K1 Escherichia coli Isolated From Bovine Mastitis
Source: Front Vet Sci. 2021 Feb 11;8:622725. doi: 10.3389/fvets.2021.622725 (PMC7904677; doi:10.3389/fvets.2021.622725)
Supplement: Supplementary file 3 [file Table_3.DOCX]

**Table S3 The background information of 69 *E. coli* isolates from bovine mastitis.**

| **Strain** | **Province** | **City** | **Phylogenetic Group** | **Serotype** | **PCR detection** | | | | | | |
| --- | --- | --- | --- | --- | --- | --- | --- | --- | --- | --- | --- |
|  |  |  |  |  | T6-Ex1 | T6-Ex2 | T6-Ex3 | T6-Ex4 | T6-Ex5 | T6-Ex6 | T6-Ex7 |
| BCE001 | Zhejiang | Xiaoshan | D | - | - | - | - | - | - | - | - |
| BCE002 | Zhejiang | Xiaoshan | A | - | + | - | - | - | - | + | - |
| BCE003 | Zhejiang | Xiaoshan | A | - | - | + | - | + | - | - | - |
| BCE004 | Jiangsu | Xuzhou | B1 | - | - | + | - | - | - | - | - |
| BCE005 | Shandong | Rizhao | A | - | + | + | - | - | + | - | - |
| BCE006 | Hebei | Langfang | B1 | - | - | - | - | - | - | - | - |
| BCE007 | Hubei | Huanggang | B1 | - | - | + | - | - | - | - | - |
| BCE008 | Jiangsu | Yancheng | A | - | - | + | - | + | - | - | - |
| BCE009 | Jiangsu | Suzhou | A | - | + | - | - | - | - | - | - |
| BCE010 | Hunan | Zhangjiakou | B1 | - | + | - | - | - | + | - | - |
| BCE011 | Hunan | Zhangjiakou | B1 | - | + | - | - | + | - | - | - |
| BCE012 | Yunnan | Dali | D | - | - | - | - | - | - | - | - |
| BCE013 | Yunnan | Dali | D | - | - | - | - | - | - | - | - |
| BCE014 | Jiangsu | Changshu | D | - | - | - | - | - | - | - | - |
| BCE015 | Shandong | Dezhou | D | - | - | - | - | - | - | - | - |
| BCE016 | Hunan | Zhangjiakou | D | - | - | - | - | - | - | - | - |
| BCE017 | Zhejiang | Xiaoshan | D | - | - | - | - | - | - | - | - |
| BCE018 | Jiangsu | Xuzhou | D | - | - | - | - | - | - | - | - |
| BCE019 | Shandong | Rizhao | D | - | - | - | - | - | - | - | - |
| BCE020 | Guangxi | Wuzhou | D | - | - | - | - | - | - | - | - |
| BCE021 | Neimenggu | Huhehaote | D | - | - | - | - | - | - | - | - |
| BCE022 | Neimenggu | Huhehaote | A | - | - | + | - | - | - | + | + |
| BCE023 | Shanghai | Nanhui | A | - | - | + | - | + | + | - | - |
| BCE024 | Shanghai | Nanhui | D | - | - | - | - | - | - | - | - |
| BCE025 | Shanghai | Nanhui | D | - | - | - | - | - | - | - | - |
| BCE026 | Shandong | Dezhou | B1 | - | - | - | - | - | - | - | - |
| BCE027 | Shandong | Dezhou | A | - | - | + | - | - | + | - | - |
| BCE028 | Heilonjiang | Heihe | A | - | - | + | - | - | - | + | - |
| BCE029 | Heilonjiang | Heihe | B1 | - | - | - | - | - | - | - | - |
| BCE030 | Heilonjiang | Heihe | D | - | - | - | - | - | - | - | - |
| BCE031 | Shanxi | Datong | D | - | - | - | - | - | - | - | - |
| BCE032 | Sichuan | Ziyang | B1 | - | - | - | - | - | - | - | - |
| BCE033 | Heilonjiang | Shuihua | B1 | - | - | + | - | + | - | - | - |
| BCE034 | Heilonjiang | Shuihua | B2 | O2 | - | - | - | - | - | - | - |
| BCE035 | Heilonjiang | Shuihua | A | - | - | - | - | - | - | - | - |
| BCE036 | Jiangsu | Xuzhou | A | - | - | - | - | - | - | - | - |
| BCE037 | Jiangsu | Xuzhou | B1 | - | - | - | - | - | - | - | - |
| BCE038 | Shanxi | Datong | A | - | - | - | - | - | - | - | - |
| BCE039 | Shanxi | Datong | B2 | O2 | + | + | - | + | + | + | + |
| BCE040 | Sichuan | Chengdu | B2 | O2 | + | + | - | + | - | - | - |
| BCE041 | Zhejiang | Xiaoshan | B1 | - | + | - | - | - | + | - | - |
| BCE042 | Jiangsu | Xuzhou | A | - | - | - | - | - | - | - | - |
| BCE043 | Shandong | Rizhao | D | - | - | - | - | - | - | - | - |
| BCE044 | Shandong | Rizhao | B1 | - | - | - | - | - | - | - | - |
| BCE045 | Zhejiang | Jinhua | B1 | - | - | - | - | - | - | - | - |
| BCE046 | Zhejiang | Jinhua | A | - | - | + | - | - | + | - | - |
| BCE047 | Zhejiang | Jinhua | A | - | - | - | - | - | - | - | - |
| BCE048 | Zhejiang | Jinhua | B2 | O2 | + | + | - | + | + | - | - |
| BCE049 | Shandong | Jining | B2 | O2 | + | + | + | + | + | + | + |
| BCE050 | Shandong | Jining | A | - | - | - | - | - | - | - | - |
| BCE051 | Shandong | Jining | A | - | - | - | - | - | - | - | - |
| BCE052 | Shandong | Jining | D | - | - | - | - | - | - | - | - |
| BCE053 | Shandong | Dezhou | B1 | - | - | - | - | - | - | - | - |
| BCE054 | Shandong | Dezhou | B1 | - | - | + | - | + | - | - | - |
| BCE055 | Shandong | Dezhou | A | - | - | + | - | - | + | - | - |
| BCE056 | Shandong | Rizhao | A | - | - | + | + | - | - | - | - |
| BCE057 | Jiangsu | Xuzhou | B1 | - | - | + | - | - | + | + | - |
| BCE058 | Shandong | Rizhao | B1 | - | + | + | - | + | + | - | - |
| BCE059 | Hebei | Langfang | A | - | - | + | + | + | - | - | - |
| BCE060 | Hubei | Huanggang | A | - | - | - | - | - | - | - | - |
| BCE061 | Hubei | Wuhan | A | - | - | - | - | - | - | - | - |
| BCE062 | Hubei | Wuhan | B2 | O1 | - | + | + | - | - | - | - |
| BCE063 | Hubei | Wuhan | B1 | - | - | + | - | + | - | + | - |
| BCE064 | Jiangsu | Xuzhou | B1 | - | - | - | - | - | - | - | - |
| BCE065 | Shandong | Rizhao | A | - | + | + | + | - | + | - | - |
| BCE066 | Hebei | Langfang | B1 | - | - | - | - | - | - | - | - |
| BCE067 | Hubei | Huanggang | A | - | - | - | - | - | - | - | - |
| BCE068 | Jiangsu | Yancheng | A | - | - | - | - | - | - | - | - |
| BCE069 | Jiangsu | Yancheng | A | - | - | - | - | - | - | - | - |
